# Hemoperitoneum detected by peritoneal dialysis as presenting symptom of cytomegalovirus-associated colitis: a case report

**DOI:** 10.1186/s12882-026-04909-x

**Published:** 2026-03-20

**Authors:** Peyman Falahat, Antolina Toma, Alexander Semaan, Sibylle von Vietinghoff, Sebastian Schwab

**Affiliations:** 1 Nephrology Section, Medical Clinic 1, University Hospital Bonn, Rheinische Friedrich-Wilhelms Universität Bonn, Venusberg Campus 1, D-53127 Bonn, Germany; 2 Institute of Virology, University Hospital Bonn, Bonn, Germany; 3 Department of Surgery, University of Bonn, Venusberg-Campus 1, 53127 Bonn, Germany

**Keywords:** Tyrosine kinase inhibitor, Checkpoint inhibitor, Renal cell carcinoma, End-stage kidney disease, Peritoneal dialysis

## Abstract

**Background:**

Immune checkpoint inhibitors and tyrosine kinase inhibitors have transformed cancer therapy also for patients with end-stage-kidney-disease.

**Case presentation:**

We here report hemoperitoneum and peritonitis in a peritoneal dialysis patient after therapies with both drug classes. The differential diagnosis included peritoneal dialysis associated peritonitis, immune checkpoint inhibitor -associated colitis, viral reactivation as well as an unrelated gastrointestinal process. Colonic resection revealed perforation, granulocytic and lymphocytic inflammation together with cytomegalovirus DNA. Symptoms responded to systemic Ganciclovir therapy.

**Conclusions:**

The case highlights the need for clinical vigilance and repeated diagnostics in the care of multimorbid individuals with unusual presentations and multidisciplinary state of the art therapies.

## Background

Immune checkpoint inhibitors (ICIs) have reshaped the treatment of advanced malignancies, delivering substantial survival benefits across tumor types. The proportion of cancer patients eligible for an ICI rose from 1.54% in 2011 to 56.55% in 2023 [1]. At the same time, autoimmune and immunosuppressive side effects need to be considered. High-dose corticosteroids are the mainstay of therapy for ICI-related adverse events, but increase susceptibility to opportunistic infections like cytomegalovirus (CMV) infection.

## Case presentation

We here report a 70-year-old man receiving peritoneal dialysis (PD). The patient’s history was remarkable for metastatic renal cell carcinoma that was diagnosed 2.3 years before the current presentation. It was initially treated with unilateral nephrectomy. The nephrectomy specimen had revealed signs of urinary congestion and chronic infection, but not glomerulonephritis. There was little proteinuria (UACR 122 mg/g creatinine) and serological workup remained negative for indicators of glomerulonephritis. PD had been commenced concomitant with the start of ICI for longstanding chronic kidney disease with an eGFR (CKD-EPI) of 37 ml/min before nephrectomy. Combined immunotherapy with nivolumab and ipilimumab was initiated 1.4 years later due to metastasis, but stopped after three months for immune-related myocarditis. Follow-up cardiac MRI showed resolution of inflammation after a three-month course of steroids. Left ventricular dysfunction persisted. Subsequent chemotherapy was performed with the tyrosine kinase inhibitor (TKI) sunitinib, which was discontinued during the present hospitalization.

During the five months before the current presentation, dialysis was complicated by recurrent lower abdominal pain, occurring at least weekly. Peritoneal dialysate was reliably clear with normal leukocyte counts, despite culture growth of *Staphylococcus epidermidis* on two previous occasions several months apart. CT imaging and ultrasound repeatedly showed nonspecific colonic wall thickening, mainly in the descending colon, without further pathology.

**Fig. 1:**
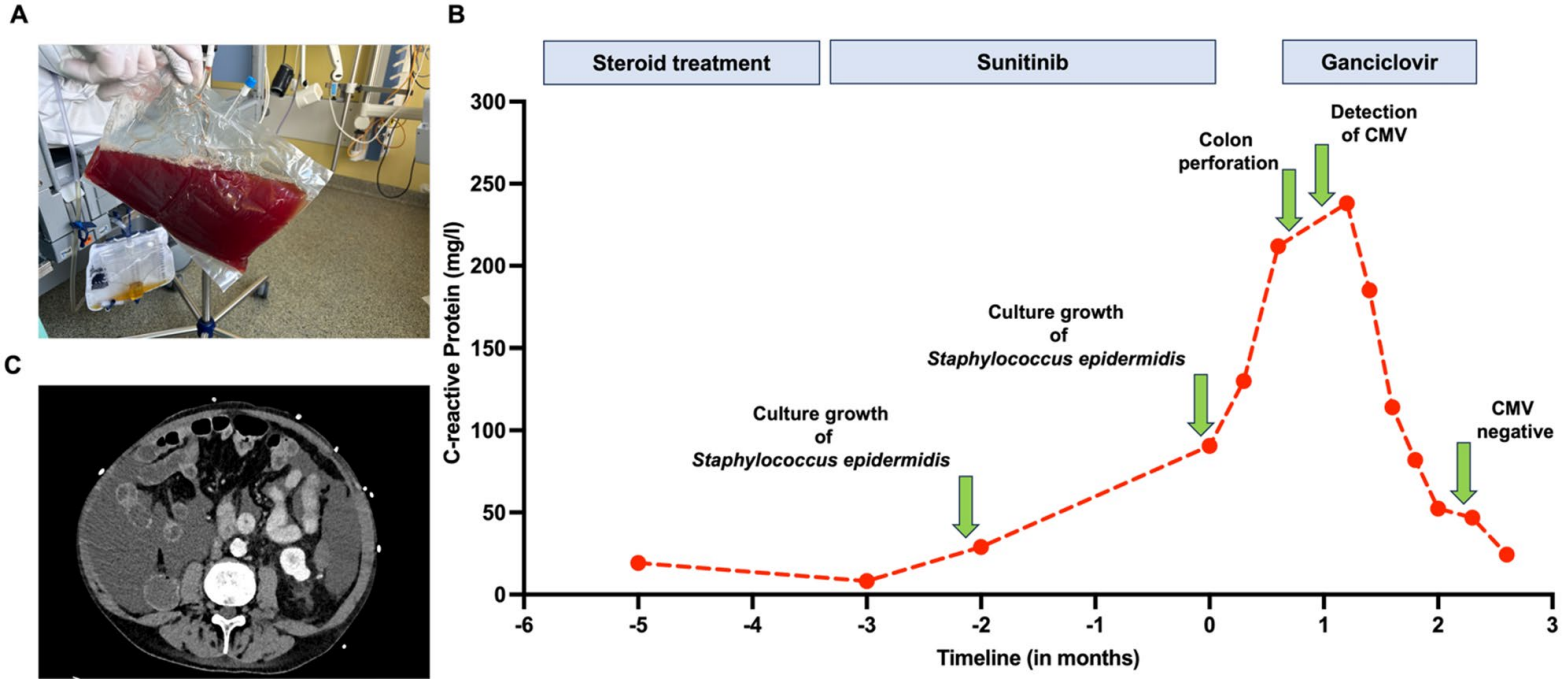
**(A)** Representative image of the peritoneal dialysate after colon perforation **(B)** CRP-values over time. Level of serum-CRP (per mg / l) as surrogate parameter for inflammation during various clinical situations and therapies. Immune checkpoint inhibitors were administered from months − 8 to -6. **(C)** CT imaging of the abdomen disclosed wall thickening of the colon and jejunal loops

The patient had been admitted seven days before the onset of hemoperitoneum because of abdominal pain. At the initial presentation, peritoneal fluid was clear and contained only 93 leukocytes/µl, 70 of which were granulocytes. However, given a single positive peritoneal culture for *Staphylococcus epidermidis*, he was treated with vancomycin for suspected PD associated peritonitis (Fig. 1B). Drug levels were in the target range. Four days after transfer to the intensive care unit for generalized tonic-clonic seizures, he developed hemoperitoneum (Fig. 1A). Laboratory analysis showed 27,000 erythrocytes/µl (from below 1000 in previous peritonitis episodes) and 4714/µl leukocytes, above 90% granulocytes. Bacterial cultures of the hemoperitoneum effluent were performed and remained negative.

Repeat abdominal imaging was performed (Fig. 1C) and revealed diffuse intestinal thickening. Upon surgical exploration and explantation of the PD catheter, covered feculent peritonitis with dense adhesions was encountered, most consistent with a contained perforation of the descending colon. A left hemicolectomy with end transverse colostomy and extensive adhesiolysis was performed. Necrotic areas and neutrophilic granulocyte infiltrations were found in the colonic specimens. Postoperatively, abdominal pain did not improve and the patient had persistent feeding difficulties. Upper gastrointestinal endoscopy revealed lymphocellular and granulocytic infiltrates, together with ulcerations. CMV-DNA was assessed by PCR and positive (6043 IU/ml) in the resected colonic tissue as well as in the upper gastrointestinal tract. Initiation of intravenous ganciclovir led to resolution of pain and systemic inflammation marker C-reactive protein (Fig. 1B).

## Discussion and conclusion

Hemoperitoneum is a prominent complication of peritoneal dialysis that can be caused by very minor bleeding [2]. Peritoneal dialysis provides a window to harmless events as well as intestinal pathology causing peritoneal bleeding that may otherwise be overlooked. In our patient, colonic perforation occurred in temporal association with tonic-clonic seizures, although a direct causal relationship cannot be inferred.

Initial presenting complaint was lower abdominal pain without clinical correlate in the peritoneal dialysate. Given the absence of bacterial growth in cultures obtained from the hemoperitoneum effluent, the previously detected *Staphylococcus epidermidis* may have represented contamination rather than persistent infection.

The frequency of gastrointestinal complaints in PD is currently underreported, according to a recent metanalysis that found their reporting in only in 19% of PD trials [3]. Among them, lower abdominal pain as reported by our patient, was common and accounted to 31% of complaints. As in our patient, diagnostic workup of these episodes is not necessarily diagnostic.

The present case also illustrates diagnostic challenges in patients with advanced malignancy. ICIs are well-recognized causes of gastrointestinal immune-related adverse events, namely autoimmune colitis that may occur in up to 30% of patients and progress to intestinal perforation [4]. The mechanism in this case appears to have been steroid-associated CMV reactivation rather than ICI-associated colitis. The patient initially developed ICI-associated myocarditis, which responded to corticosteroids. However, subsequent immunosuppression likely predisposed to CMV colitis with eventual perforation. This emphasizes the importance of considering opportunistic infections such as CMV in patients receiving prolonged immunosuppressive therapy for ICI toxicities. Given the patient’s exposure to sunitinib, we considered a VEGF-inhibitor–related mechanism for the abdominal symptoms. Sunitinib commonly causes gastrointestinal adverse events [5], but intestinal perforation has only been reported in a limited number of case reports. While a contributory role of sunitinib in lowering the threshold for perforation cannot be excluded, the temporal relationship to prior ICI exposure, occurrence of abdominal pain before initiation of sunitinib treatment and histological findings of CMV infection support CMV colitis as the most plausible explanation.

In peritoneal dialysis, peritonitis subsequent to CMV- colitis with bacterial superinfection is currently, to the best of our knowledge, limited to one case of a patient without concomitant immunosuppression or chemotherapy [6]. Our case may thus contribute to raise awareness to detection of complications of contemporary tumor therapy in this patient population.

## Data Availability

The data underlying this article will be shared on reasonable request to the corresponding author.

## Abbreviations

CMV: Cytomegalovirus
GFR: Glomerular filtration rate
ICI: Immune checkpoint inhibitor
PD: Peritoneal dialysis
UACR: Urine albumin creatinine ratio
TKI: Tyrosin kinase inhibitor
